# SGLT2 inhibitors: from glucose-lowering to cardiovascular benefits

**DOI:** 10.1093/cvr/cvae047

**Published:** 2024-03-08

**Authors:** Alberto Preda, Fabrizio Montecucco, Federico Carbone, Giovanni G Camici, Thomas F Lüscher, Simon Kraler, Luca Liberale

**Affiliations:** Department of Clinical Cardiology, IRCCS San Raffaele Hospital, Vita-Salute San Raffaele University, Milan, Italy; First Clinic of Internal Medicine, Department of Internal Medicine, University of Genoa, 6 viale Benedetto XV, 16132 Genoa, Italy; IRCCS Ospedale Policlinico San Martino Genoa—Italian Cardiovascular Network, Genoa, Italy; First Clinic of Internal Medicine, Department of Internal Medicine, University of Genoa, 6 viale Benedetto XV, 16132 Genoa, Italy; IRCCS Ospedale Policlinico San Martino Genoa—Italian Cardiovascular Network, Genoa, Italy; Center for Molecular Cardiology, University of Zürich, Schlieren, Switzerland; Department of Research and Education, University Hospital Zurich, Zurich, Switzerland; Center for Molecular Cardiology, University of Zürich, Schlieren, Switzerland; Royal Brompton and Harefield Hospitals and Imperial College and King’s College, London, United Kingdom; Center for Molecular Cardiology, University of Zürich, Schlieren, Switzerland; Department of Internal Medicine, Cantonal Hospital Baden, Baden, Switzerland; First Clinic of Internal Medicine, Department of Internal Medicine, University of Genoa, 6 viale Benedetto XV, 16132 Genoa, Italy; IRCCS Ospedale Policlinico San Martino Genoa—Italian Cardiovascular Network, Genoa, Italy

**Keywords:** SGLT2 inhibitors, diabetes, heart failure, inflammation, oxidative stress, autophagy, mitochondria, endothelial function

## Abstract

An increasing number of individuals are at high risk of type 2 diabetes (T2D) and its cardiovascular complications, including heart failure (HF), chronic kidney disease (CKD), and eventually premature death. The sodium-glucose co-transporter-2 (SGLT2) protein sits in the proximal tubule of human nephrons to regulate glucose reabsorption and its inhibition by gliflozins represents the cornerstone of contemporary T2D and HF management. Herein, we aim to provide an updated overview of the pleiotropy of gliflozins, provide mechanistic insights and delineate related cardiovascular (CV) benefits. By discussing contemporary evidence obtained in preclinical models and landmark randomized controlled trials, we move from bench to bedside across the broad spectrum of cardio- and cerebrovascular diseases. With landmark randomized controlled trials confirming a reduction in major adverse CV events (MACE; composite endpoint of CV death, non-fatal myocardial infarction, and non-fatal stroke), SGLT2 inhibitors strongly mitigate the risk for heart failure hospitalization in diabetics and non-diabetics alike while conferring renoprotection in specific patient populations. Along four major pathophysiological axes (i.e. at systemic, vascular, cardiac, and renal levels), we provide insights into the key mechanisms that may underlie their beneficial effects, including gliflozins’ role in the modulation of inflammation, oxidative stress, cellular energy metabolism, and housekeeping mechanisms. We also discuss how this drug class controls hyperglycaemia, ketogenesis, natriuresis, and hyperuricaemia, collectively contributing to their pleiotropic effects. Finally, evolving data in the setting of cerebrovascular diseases and arrhythmias are presented and potential implications for future research and clinical practice are comprehensively reviewed.

## 1. Introduction

The surge in incidence and prevalence of type II diabetes mellitus (T2D) challenges healthcare systems around the globe. Indeed, the number of diabetic patients is expected to almost double from 382 million in 2013 to 592 million by 2035,^1^ putting an increasing proportion of individuals at increased cardiovascular (CV) risk. In fact, patients with T2D are at two- to three-fold increased risk of developing CV disease,^2^ the leading cause of morbidity and mortality in developed countries and beyond.^3^ Concurring with the increased CV risk, T2D is a major driver of chronic kidney disease (CKD) and accelerates its progression to end-stage renal disease (ESRD) ultimately leading to renal replacement therapy.^4^

Considering the above, glycaemic control intertwined with multidisciplinary CV risk reduction remains therapeutic hallmarks of T2D management. Over the last few decades, our armamentarium for the medical treatment of T2D expanded considerably, with randomized evidence on sodium–glucose co-transporter-2 (SGLT2) inhibitors (SGLT2i) and glucagon-like peptide-1 receptor agonists (GLP-1RA) providing convincing evidence for the prevention of CV complications. SGLTi, (also referred to as gliflozins), effectively reduce blood glucose and glycated haemoglobin levels among patients with T2D without inducing hypoglycaemia. In addition, gliflozins—in contrast to traditional antidiabetics—prevent heart failure (HF) hospitalizations and improve renal function irrespective of T2D with negligible adverse effects.

Accordingly, GLP-1RA or SGLT2i are recommended by the *2023 ESC Guidelines for the Management of Cardiovascular Disease in Patients with Diabetes* as first-line therapy for T2D patients at high CV risk, such as those with established atherosclerotic cardiovascular diseases (ASCVD), CKD or HF.^5^ The use of GLP-1RA and SGLT2i increased markedly in recent years, with SGLT2i now leading prescription rates.^6^ In addition to their convenient oral route of administration, SGLT2i reduce the risk of the combined endpoint of CV death and hospitalizations in patients with HF irrespective of ejection fraction,^7,8^ and are accordingly recommended by both European and American guidelines.^9–11^ Herein, we aim to provide a translational overview of SGLT2i, covering both experimental and clinical evidence, moving from mechanisms studied in preclinical models to the clinical setting across the broad spectrum of CV disease.

## 2. Mechanisms underlying glucose-lowering

SGLT2 expression is mainly confined to the proximal tubule of the nephron, contributing to roughly 90% of the kidney's capacity to reabsorb glucose.^12^ While transepithelial glucose uptake is an active Na^+^/K^+^ ATPase-dependent process that is driven by the sodium gradient across the brush-border membrane,^13^ intracellularly accumulated glucose exits the tubular cells via glucose transporter-2 towards the interstitial fluid and the peritubular capillary, making SGLT2 an attractive therapeutic target to reduce glucose reabsorption from urine back to the circulation. SGLT2i development was stimulated by the identification of phlorizin, a glucoside typically found in the root bark of fruit trees and known to block sugar transport in several tissues, including the kidney and small intestine.^14,15^ Initial attempts to study its glucose-lowering effects in diabetic rats confirmed that phlorizin-induced glycosuria indeed normalizes plasma glucose levels and restores insulin sensitivity without inducing hypoglycaemia.^16^ While the expression of low-capacity but high-affinity SGLT1 is not confined to one particular organ or tissue type, that of high-capacity, but low-affinity SGLT2 is almost exclusively limited to the epithelium of the proximal tubule, responsible for the reabsorption of urinary glucose.^17^ Importantly, among healthy individuals, nearly 100% of the glucose that is filtered by the renal glomerulus is subsequently reabsorbed.^18^ However, when plasma glucose levels exceed a certain threshold (∼180 mg/dL or 10 mmol/L), glucose reabsorption capacity declines, while urinary glucose excretion increases.^19^

European Medicines Agency (EMA)- and Food and Drug Administration (FDA)-approved SGLT2i such as canagliflozin, dapagliflozin, empagliflozin, and ertugliflozin share important pharmacokinetic characteristics, namely, rapid oral absorption, long half-life (allowing once-daily administration), wide hepatic metabolism (mainly via glucuronidation to inactive metabolites) and, most importantly, few clinically relevant drug–drug interactions (except an increased risk of hypoglycaemia with concomitant administration with insulin and/or sulphonylureas).^20^ Placebo-controlled studies in patients with T2D demonstrated a consistent, albeit modest glucose-lowering effect of SGLT2i compared to other glucose-lowering agents, including sulfonylureas and GLP1-RA, with a relative reduction in HbA1c levels of 0.6% up to 1.0% over 12–78 weeks^21–23^ and attenuated efficacy in patients with CKD. Specifically, comparative reports with sulfonylureas (i.e. glimepiride and glipizide) are not always consistent and suggest different effects for different molecules with empagliflozin and canagliflozin showing higher efficacy.^24–26^ Whilst GLP-1Ras such as semaglutide show a higher glucose-lowering effect with a mean difference in HbA1c% levels of up to 0.6% at 26 months compared to SGLT2i.^27,28^ Finally, gliflozins achieve comparable HbA1c% reductions relative to dipeptidyl peptidase-4 (DPP4) inhibitors.^29–31^ Major side-effects observed in randomized clinical trials (RCTs) include female genital mycotic infections, urinary tract infections (including urosepsis and pyelonephritis), nausea, constipation, and ketoacidosis.^32^

## 3. Experimental evidence of their cardiovascular benefits

Gliflozins’ actions involve cellular and molecular mechanisms at vascular, renal, cardiac, and systemic levels, with their cardiorenal effects most prominently contributing to their CV benefits. While some are related to the prime action of SGLT2 on renal tubules, gliflozins exert effects that act independently of this pathway. In support of this notion, empagliflozin exerts cardioprotective effects in mice deprived of renal SGLT2 expression.^33^ To disentangle the key mechanisms by which SGLT2i confer CV/-renal benefits, we herein provide a streamlined overview of their mechanism of action, focusing on cardiac, renal, vascular, and systemic levels, between which a strong interplay exists.

### 3.1 Vascular effects

**Figure 1:**
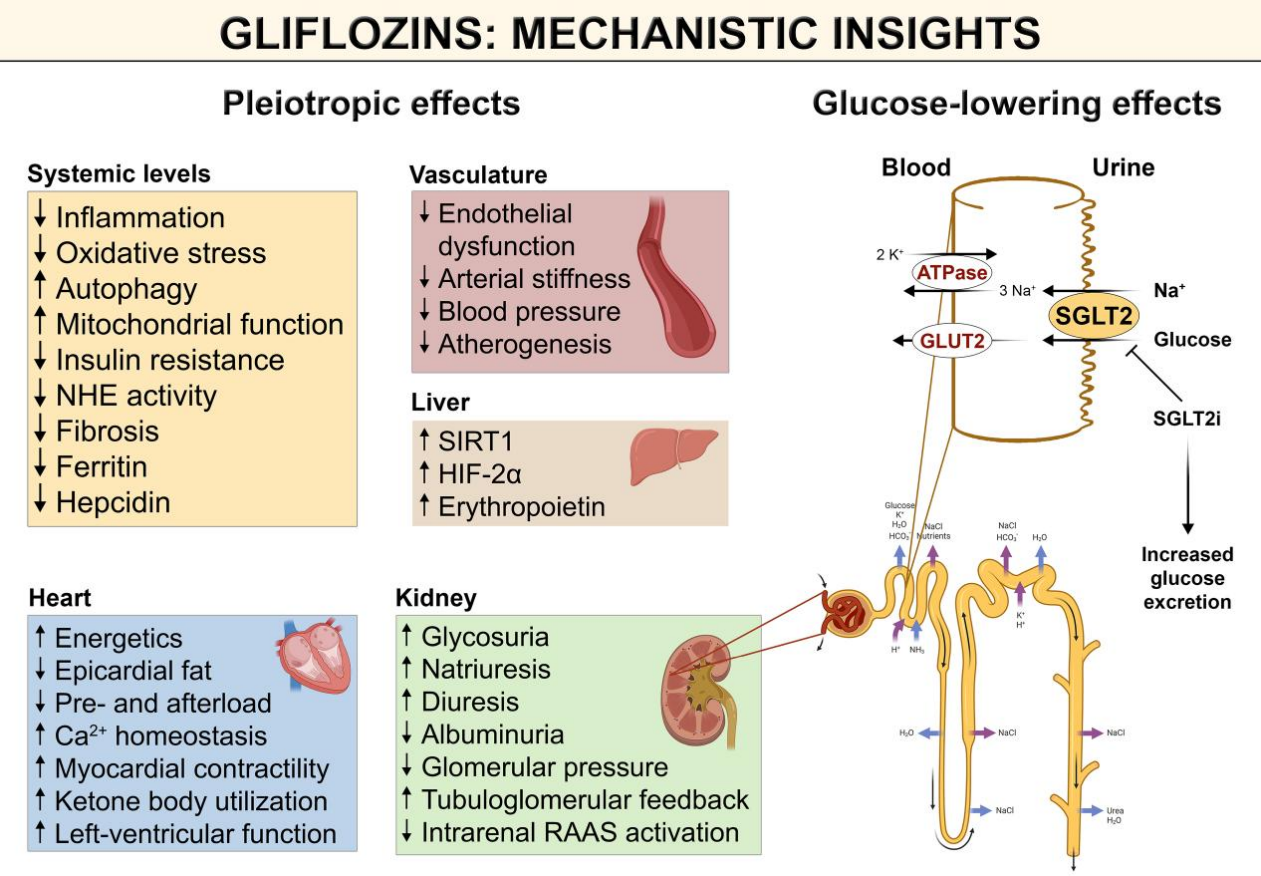
Mechanistic insights into the role of gliflozins at systemic, vascular, cardiac, and renal levels. Preclinical evidence suggests a potential beneficial effect of gliflozins in preclinical models mimicking renal, cardiac, and vascular pathologies. Involving a variety of mechanisms independently of their glucose-lowering effects, SGLT2 inhibition reduces inflammatory signalling, mitigates oxidative stress, improves autophagic flux, and enhances mitochondrial function, therethrough contributing to their cardiorenal/-vascular benefits. SGLT2, sodium-glucose transporter 2; NHE, sodium/hydrogen exchanger. Created with content provided by BioRender.com.

A large body of experimental evidence suggests that SGLT2i exert vasculoprotective effects involving mechanisms that may act independently of their glucose-lowering properties (*Figure 1*).^34^ The beneficial effects of gliflozins on endothelial function are mediated by (i) increased nitric oxide (NO) availability, (ii) reduced oxidative stress, (iii) alleviated microvascular dysfunction, (iv) preserved mitochondrial function, (v) reduced inflammation, and (vi) reinstalled energy homeostasis. These mechanisms are closely intertwined with each other; thus, the relevance of these key mechanism towards clinical benefit is, at present, difficult to judge. Yet, their regulatory properties on both mitochondrial function and cellular energy homeostasis might be of particular relevance in driving their CV benefits.^35^

#### 3.1.1 NO-mediated attenuation of oxidative stress

NO is the most potent mediator of endothelial homeostasis and critically regulated by SGLT2i. Indeed, in vascular cells, SGLT2i increase adenosine monophosphate (AMP)-activated protein kinase (AMPK) activity and enhance endothelial NO synthase (eNOS) phosphorylation at Ser1177 site with direct effects on vascular function.^36–38^ While angiotensin II induces SGLT2 expression in mice and accelerates oxidative stress involving nicotinamide adenine dinucleotide phosphate (NADPH) oxidase activation,^39,40^ empagliflozin suppresses activation of NADPH oxidase thus protecting against oxidative stress and in turn endothelial senescence and dysfunction.^41,42^ Moreover, empagliflozin may reduce the levels of cyclic guanosine monophosphate(cGMP)-dependent kinase (cGK-I), a negative regulator of the effect of NO that contributes to vascular dysfunction.^43,44^ While canagliflozin exhibits similar pro-NO effects, these were intriguingly mainly driven by NOX2- and NOX4-mediated mechanisms.^45–47^ The aforementioned effects directly relate to improved vasorelaxation in several animal models of impaired vascular function.

The link between NO bioavailability and oxidative stress has been reviewed elsewhere.^48^ Briefly, NO bioavailability also directly depends on oxidative stress as NO and O_2_^−^ rapidly interact to form the toxic peroxynitrate. Downstream effectors of Ang II, SGLT1-, and 2 exert significant pro-oxidative effects in the vasculature. Consequently, both empagliflozin and dapagliflozin protect the endothelium from intracellular ROS production and in turn improve NO bioavailability, mainly mediated by increased levels of antioxidants such as glutathione, glutathione peroxidase, and superoxide dismutase.^49,50^ More recently, STAT-3 pathway activation and increased Y(705)STAT-3 dimers together with Na^+^/H^+^ exchanger (NHE) 1 and NADPH oxidase have been discussed as possible mediators of their beneficial effect on intracellular ROS production.^51,52^

#### 3.1.2 Preservation of mitochondrial function

Mitochondria sense environmental stress and mediate cellular adaptation by modulating energy production and reactive oxygen species (ROS) formation (thereby looping back to the mechanisms outlined above) with direct effects on endothelial growth, proliferation, and senescence.

Of interest, SGLT2i are thought to prevent mitochondrial DNA damage by modulating the physiological process of mitochondrial fission by restoring the AMP-to-adenosine triphosphate (ATP) ratio, increasing AMPK activation and reducing Drp1 phosphorylation.^53^ Also, by improving energy production they preserve respiratory chain function, stabilize their membrane potential, reduce ROS synthesis, and the opening of mitochondrial permeability transition pore (PTP) channels with direct effect on cell viability.^54^ Reduced levels of oxidative stress preserve mitochondrial DNA and reduce mitochondrial fission 1 (Fis1) protein activation, again modulating mitochondrial fission.^55^ Also, dapagliflozin was reported to facilitate the opposite mechanism of mitochondrial fusion by upregulating Opa1 and Mfn2.^56^

Furthermore, SGLT2i were shown to maintain autophagic flux (i.e. a measure of autophagic degradation activity) and promote mitochondrial health.^57–61^ Importantly, aberrant autophagic flux is implicated into the build-up of atherosclerotic plaques,^62,63^ with both empagliflozin and dapagliflozin enhancing autophagy in cardiometabolic disease models via the activation of nutrient-sensing pathways such as the mTOR, sirtuin1 (SIRT1), and hypoxia-inducible factor (HIS) pathway.^64–66^

#### 3.1.3 Alleviation of microvascular dysfunction

Coronary microvascular dysfunction encompasses structural and functional alterations perturbing coronary microcirculation homeostasis, thus contributing importantly to the pathogenesis of several forms of HF, including HF with preserved ejection fraction (HFpEF).^67^ In this regard, it is noteworthy that gliflozins emerged as alleviators of microvascular dysfunction during several conditions, including diabetic and pro-inflammatory states,^50,68^ reviewed in detail by Dimitriadis *et al*.^69^ For instance, in ob/ob^−/−^ mice, empagliflozin-mediated SGLT2 inhibition over 10 weeks improved coronary microvascular function reflecting into improved cardiac contractile performance.^68^ Similarly, Juni *et al*. have shown that empagliflozin alleviates microvascular dysfunction by reinstating endothelial NO delivery through reduced tumor necrosis factor (TNF)α-induced ROS accumulation, thereby positively influencing cardiomyocyte contraction and relaxation.^50^ In line, in an *in vitro* model of CKD, empagliflozin restored microvascular endothelial NO-dependent cardiomyocyte function upon exposure to uraemic serum.^49^ Collectively, these experimental data highlight the interaction of the endothelium and myocardium, with SGLT2i likely exerting their beneficial effects on cardiac function involving, at least in part, endothelium-dependent mechanisms, as outlined above.

#### 3.1.4 Inhibition of inflammation

**Figure 2:**
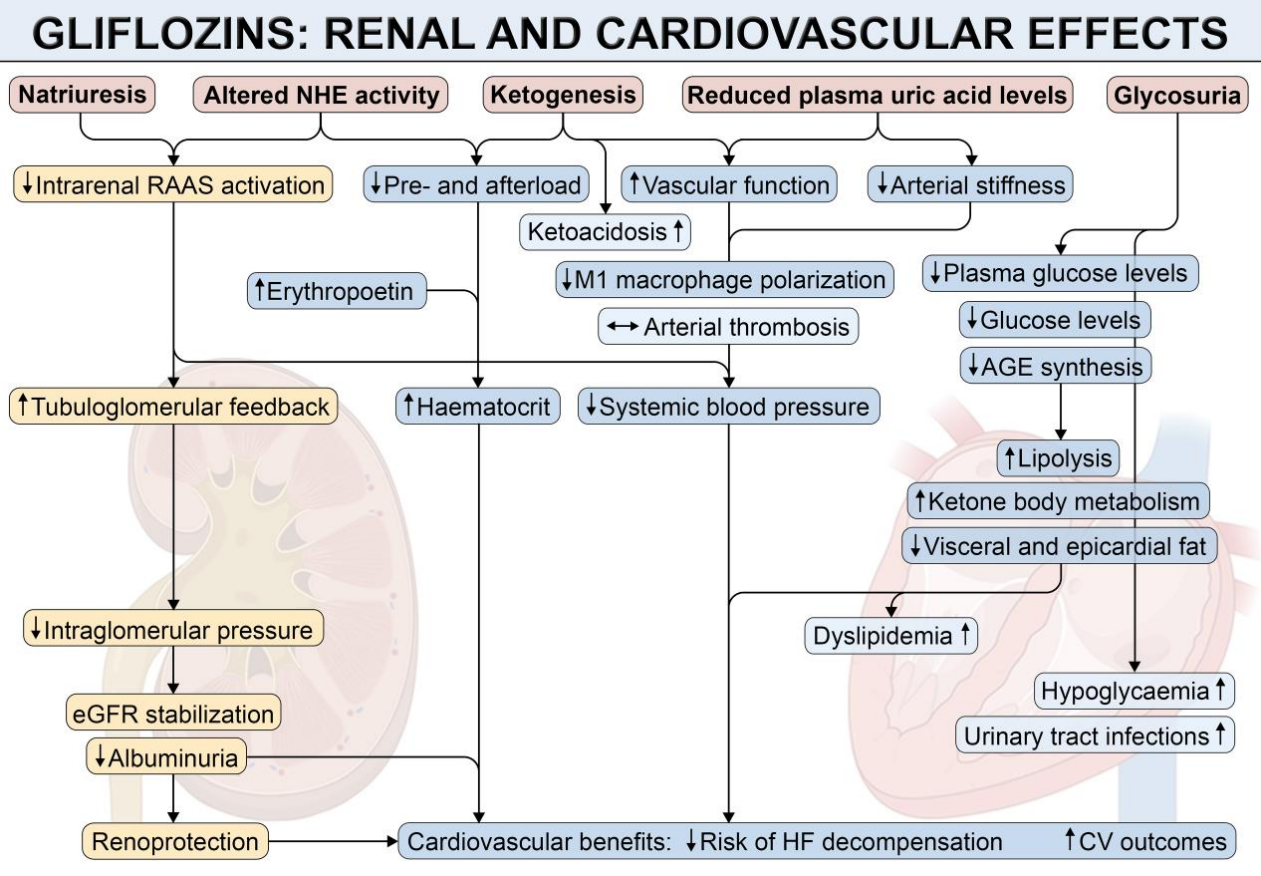
Systemic effects of gliflozins. Gliflozins exert natriuretic effects, impact tissue sodium handling, increase ketogenesis, and reduce both plasma uric acid and glucose levels, leading to a variety of beneficial effects which collectively results in improved renal and CV outcomes. Note that neutral or negative effects are highlighted in bright blue, with lower urinary tract infections ranking among the most frequently observed side-effects. Created with content provided by BioRender.com.

SGLT2i show anti-inflammatory properties. Indeed, treatment with gliflozins reduced the activation of NF-kB transcription factor in several models of cardiometabolic diseases with reduced levels of monocyte chemoattractant protein-1 (MCP-1), TNFα, interleukin-6 (IL-6), and high-sensitivity C-reactive protein.^58,70,71^ Also, SGLT2 reduced the activation of NLRP3 inflammasome, expression of intercellular adhesion molecule (ICAM)-1, vascular cell adhesion molecule (VCAM)-1, P-, and E-selectin with reduced inflammatory cell recruitment and activation.^72,73^ (*Figure 2*). By doing so, SGLT2i improve endothelial integrity and barrier function.

Given the association between endothelial inflammation and production of prothrombotic factors such as tissue factor and plasminogen activator inhibitor-1 (PAI-1), different experimental studies investigated a potential role for gliflozins in mediating arterial thrombosis, a dreadful but common complication of atherosclerosis. While a small pilot study yielded promising results, we have previously shown their neutral effects on arterial thrombus formation, a finding that is in line with landmark trials.^56,74^

#### 3.1.5 Restoration of energy homeostasis

The beneficial metabolic effects of SGLT2i also include reduced insulin resistance, a common denominator of cardiometabolic diseases. Importantly, owing to increased urinary glucose excretion, SGLT2i administration triggers a loss of ∼200–250 kcal per day.^75^ This leads to a decline in both visceral and subcutaneous adipose tissue, with preservation of lean tissue mass and a loss of extracellular fluid, though only transiently.^76^ In line, RCTs report a reduction of about 2 kg as compared to placebo,^77,78^ an effect that notably plateaus after 6 months.^79^ Mechanisms causing bodyweight reduction differ between SGLT2i and GLP-1RA, as the latter promote mainly satiety and fullness due to delayed gastric emptying thereby reducing calorie intake.^80^

Gliflozins promote oxidation of both glucose and long fatty acids independently of insulin levels with a modest increase in ketone body production (as reflected by higher plasma levels),^81,82^ with this metabolic shift likely representing an important contributor to their CV benefits. Furthermore, SGLT2i modulate extracellular volume, an effect likely mediated only partially by their natriuretic and glycosuric effects as their increase in diuresis is transient. Inhibition of renin-angiotensin-aldosterone system (RAAS) and subsequent blood pressure reduction, body weight reduction, improved glycaemic control, and vascular function are overall, mechanisms acting in longer term which might explain this effect.^78,83–86^ Also, by increasing glycosuria, SGLT2i reduce urate absorption in the proximal convoluted tubule,^87^ leading to a persistent reduction of circulating uric acid.^88^ Notably, uric acid is pro-inflammatory (e.g. activation NLRP3 among others), it fosters fibrosis within the vascular wall,^89^ increases apoptosis of endothelial cells,^90^ and eventually depletes endothelial NO levels.^91^ Nonetheless, the causal role of urate as a modulator of CV outcomes is still heavily debated. Indeed, the association between genetic markers of hyperuricaemia and CV outcomes is only weak in magnitude^92^ with allopurinol showing neutral effects on CV outcomes in the large ALL-HEART study.^93^

### 3.2 Renal effects

These vasculoprotective roles are of fundamental relevance for the understanding of SGLT2i-mediated renoprotection.^94^ Indeed, renovascular oxidative stress drives the initiation and progression of CKD with more recent evidence implicating the proximal tubule in the pathogenesis of diabetic nephropathy.^95,96^ Accordingly, SGLT2 expression is mainly confined to proximal tubular cells, a cell population rich in mitochondria and centrally involved in ROS production.^97^ At the renal level, preclinical studies reported that SGLT2 inhibition may be linked to reduced synthesis of pro-inflammatory and -fibrotic mediators (TNFR1, IL-6, MMP7, and FN1),^98^ blunted expression of deleterious toll-like receptor-4 (TLR-4) and attenuated activation of nuclear factor kappa B (NF-κB) culminating in an overall anti-fibrotic effect.^99^ Mechanistically, human proximal tubular epithelial cells treated with SGLT2i showed reduced collagen IV and α-smooth muscle actin (α-SMA) expression, but an increase in protective STAT1 and transforming growth factor-β1 (TGF-β1).^100^ Furthermore, renoprotective effects of gliflozins also include a reduction in volume overload, lower intraglomerular blood pressure due to tubule-glomerular feedback modulation as well as reduced sympathetic and RAAS activation with subsequent natriuresis. Indeed, SGLT2i exert natriuretic effects through NHE3 inhibition,^101–105^ a protein displaying structural overlaps with SGLT2.^106,107^ NHE3 is mainly expressed on the apical surface of renal epithelial cells, where it regulates sodium reuptake following glomerular filtration.^107,108^ With NHE3 being upregulated by RAAS hyperactivation in patients with HF and T2D, renoprotective effects of SGLT2i are thought to be, at least in part, NHE-dependent. Yet, as noted above, their natriuretic and volume depletion effects occur transiently without resulting in neurohormonal activation, kidney dysfunction, or electrolyte abnormalities as observed with loop diuretics.^109^ Bourlaug and Testani recently suggested that, differently from traditional diuretics, SGLT2i’ effect on plasma volume occurs due to an adjustment in the volume setpoint definition facilitating increased diuresis in hypervolemic patients only.^110^ This elegant concept implies that the mechanism depends on the location of SGLT2 in the proximal tubule, resulting in increased delivery of salt-rich fluid at the level of the *macula densa* with suppressed RAAS activation thus driving increased Na^+^ and water reabsorption when needed in the remaining parts of the nephron.^110^

Finally, SGLT2i are associated with increased haematocrit values, an effect that is partially due to their diuretic properties but also relates to enhanced erythropoiesis.^111^ How SGLT2i increase red blood cell production remains to be fully delineated. Among the different hypotheses, SGLT2i may (i) rejuvenate erythropoietin-producing cells by increasing renal oxygenation, (ii) cause localized hypoxia at the tubule stimulating erythropoietin production, (iii) increase iron utilization through hypoxia inducible factor (HIF)-2α; or (iv) enhanced erythropoietin gene transcription by increased SIRT1.^112^

### 3.3 From systemic effects towards cardioprotection

Most of the cardioprotective effects of SGLT2i on cardiac structure and function are mediated by their systemic haemodynamic and metabolic effects. Indeed, vascular effects are related to blood pressure reduction by osmotic diuresis and natriuresis, attenuation of arterial stiffness as well as improved endothelial function.^86^ Moreover, body weight reduction and a decline in total plasma volume by increased glucose excretion and enhanced erythropoiesis contribute to improved cardiac output.^76,113^ In the failing heart, compromised energy and redox balance along with perturbations of ion regulatory mechanisms occur early during the disease process, through which myocardial remodelling and contractile dysfunction are promoted.^114–117^ Dysfunctional mitochondria lead to compromised energy and redox balance fostering contractile dysfunction and apoptotic cell death of cardiomyocytes.^118,119^ Increased NADPH oxidase activity and advanced glycation end products (AGEs) generation are principal causes of oxidative stress in myocardial cells of T2D patients.^120^ SGLT2i counteract oxidative stress, induce M2 macrophage polarization and reduce TGF-β production thereby blunting progression of myocardial fibrosis.^121,122^ A clinical study is presently exploring whether this translates into improved myocardial strain, fibrosis, and inflammation as assessed by cardiac MRI (NCT03782259). Other anti-inflammatory effects may be mediated by reduction of epicardial adipose tissue,^123^ changes of apolipoproteins profile (i.e. increase of LDL and HDL and decrease of triglycerides),^124^ and amelioration of liver steatosis.^125^ Failing cardiomyocytes also exhibit altered cellular energy sensor function as evidenced by compromised AMPK function,^126,127^ collectively driving the transition towards a catabolic phenotype involving increased glucose uptake and accelerated glycolysis.^128,129^ By increasing pancreatic glucagon release and suppressing insulin (although this finding is controversial and has not been consistently replicated in all studies),^130^ SGLT2i might also increase ketone bodies production which are well-known alternative energy sources in failing hearts.^129,131,132^ Mechanistically, this may explain the higher risk of euglycemic ketoacidosis reported in some clinical studies.^133^

**Figure 3:**
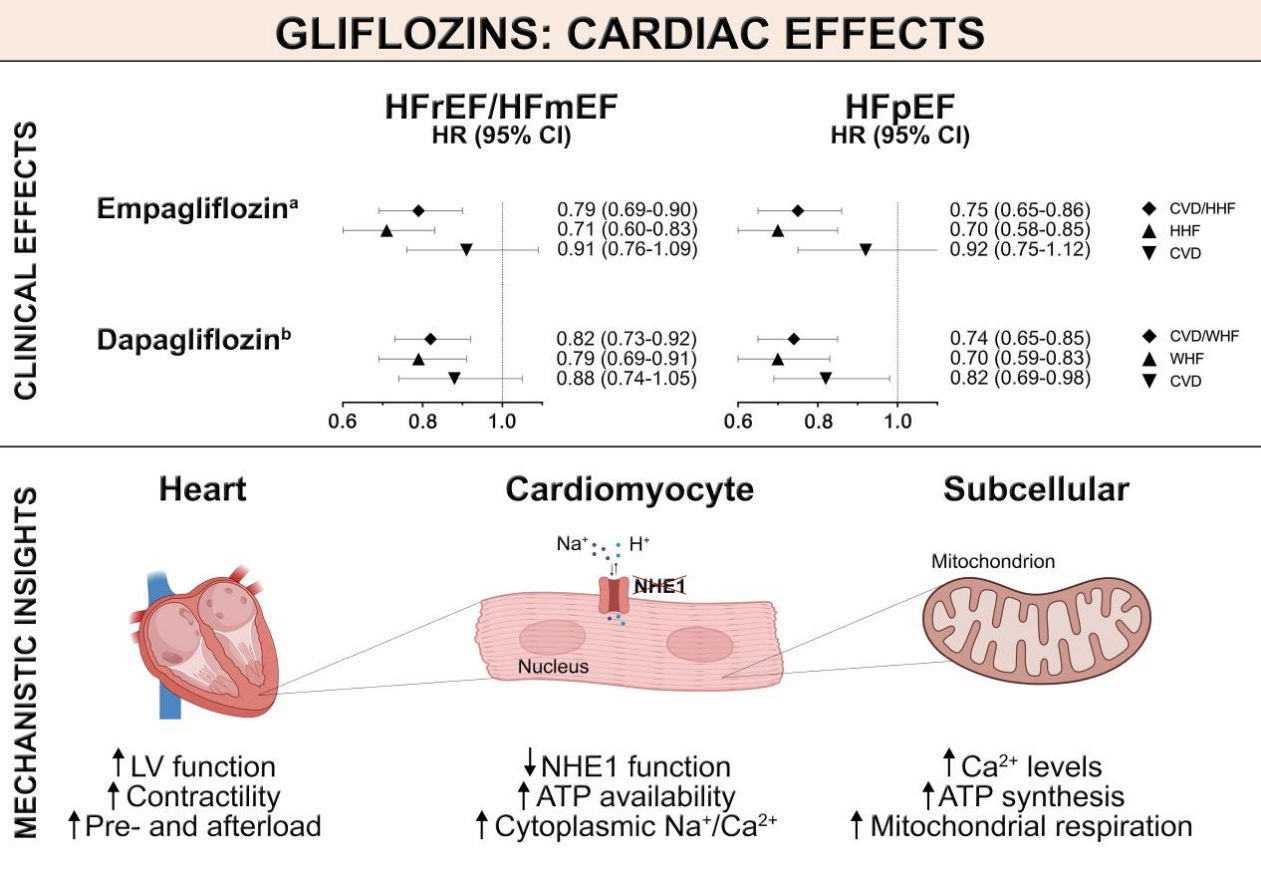
The cardiac effect of gliflozins across the HF spectrum. Empagliflozin and dapagliflozin reduce adverse CV outcomes in patients with HF independently of the ejection fraction. The upper panel shows the clinical benefits of SGLT2 inhibition, with treatment effects shown as hazard ratios (HR) along with their 95% confidence intervals (CI). The lower panel provides insights into potential mechanisms underpinning cardiac benefits. ATP, Adenosine triphosphate; CD, cardiovascular death; CI, confidence interval; HFmrEF, heart failure with mildly reduced ejection fraction; HFpEF, heart failure with preserved ejection fraction; HFrEF, heart failure with reduced ejection fraction; HHF, hospitalization for heart failure; HR, hazard ratio; LV, left-ventricular; NHE, sodium/hydrogen exchanger; WHF, worsening of heart failure. Created with content provided by BioRender.com. ^a^Refers to the EMPEROR-Reduced Trial^152^ and EMPEROR-Preserved Trial.^153^  ^b^Refers to the DAPA-HF Trial^154^ and DELIVER Trial.^155^

Importantly, ion channel regulating mechanisms sit at the crossroads of housekeeping mechanisms, myocardial remodelling, and contractile (dys-)function and thus may open novel exciting therapeutic avenues.^114,134–137^ In this regard it is interesting to note that SGLT2i improve myocardial Ca^2+^ homeostasis by modulating the activity of cardiac-specific NHE1 and in turn mitochondrial respiration, thereby blunting the decline in contractility.^138–140^ (*Figure 3*) Also, in cardiomyocytes, SGLT2i may facilitate contraction and relaxation cycles by blunting the late inward sodium current and calcium–calmodulin–dependent protein kinase II.^139^ Moreover, SGLT2i share characteristics of caloric restriction mimetics not only through their induction of glucosuria, but also by modulating cellular energy sensors such as AMPK and sirtuins.^64,141,142^ This is of particular relevance since AMPK activation mitigates oxidative stress, blunts pro-inflammatory signalling, and confers resilience to cellular stress, thus exerting beneficial metabolic effects.^143^ The beneficial metabolic reprogramming provided by SGLT2i at cardiorenal levels has been recently reviewed elsewhere.^144^ At the functional level, enhanced activity of the AMPK pathway attenuates HF-related phenotypes,^145^ while regulating vascular tone via suppression of inflammatory signalling.^146^ Importantly, cardioprotective effects of SGLT2i following hypoxia/reoxygenation injury were also reported, independently of the presence or absence of diabetes and exposure to different AGE concentrations.^147^ A recent meta-analysis of preclinical studies provided evidence that SGLT2i have the potential to reduce myocardial infarct size (by 33% in infarct size/area at risk), independently of the presence of diabetes.^148^ Of interest, the heart-sparing effect of empagliflozin in the context of myocardial infarction may be at least in part independent of SGLT2 effects as shown in mice lacking this protein by isolated Langendorff-perfused heart model.^33^ In addition, empagliflozin proved effective in ameliorating adverse cardiac remodelling in a nondiabetic porcine model of HF predominantly by reinstating myocardial energetics, blunting histological and molecular abnormalities of cardiac remodelling, improving systolic and diastolic function and reducing left ventricle (LV) stiffness.^149–151^

## 4. Clinical evidence across the spectrum of CV diseases

Innovative RCTs made substantial contributions to our understanding of the CV benefits of SGLT2i in T2D, HF, CKD, and beyond. Although initially developed and approved for their glucose-lowering effects, SGLT2i are increasingly prescribed due to their CV/-renal benefits, with glucose-lowering deemed to represent a beneficial side-effect.

### 4.1 Gliflozins and CV outcomes

Contemporary meta-analyses suggest that SGLT2i reduce HF hospitalizations and reduce the risk of worsening renal function ameliorating renal outcomes in patients with T2D and/or HF.^8,156–159^ DECLARE-TIMI 58,^160^ EMPA-REG OUTCOME,^161^ and CANVAS Program^162^ demonstrated the superiority of empagliflozin, dapagliflozin, and canagliflozin vs. placebo in patients with T2D, CV risk factors or established atherosclerotic disease (ASCVD) (*Table 1*). Among recruited patients, the mean age was roughly 65 years, 1/3 were females, 10% with HFrEF and 25% with CKD (only 7.4% in DECLARE-TIMI 58). Median follow-up ranged from 2.4 years in CANVAS to 4.2 years in DECLARE-TIMI 58. The primary endpoint was a three-point MACE (composite of CV death, non-fatal acute myocardial infarction (AMI) and non-fatal stroke) while additional endpoints comprised hospitalization for HF (co-primary in DECLARE-TIMI 58). SGLT2 inhibition is associated with a relative risk (RR) reduction of 14%, mainly driven by a dramatic reduction in hospitalizations for HF (RR of 24%). Of note, only empagliflozin provided a significant reduction in CV death (RR of 38%), with neutral effects being observed for non-fatal AMI and non-fatal stroke. Two additional trials added important evidence for the efficacy of SGLT2i in T2D, namely the VERTIS CV and CREDENCE trials.^163,164^ VERTIS CV^163^ evaluated ertugliflozin vs. placebo for the three-point MACE showing a benefit for HF hospitalization (RR −30%). The CREDENCE trial^164^ enrolled patients with T2D-related CKD. Although the incidence of MACE or hospitalization for HF were not primary outcomes, a robust attenuation of both was detected (RR −20% and −39%, respectively). Notably, in this selected population, canagliflozin effectively reduced CV death, even if only borderline significant [hazard ratio (HR), 0.78; 95% CI, 0.61–1.00; *P* = 0.05].

**Table 1.** Key clinical trials (in terms of findings and number of patients enrolled) of SGLT2i in patients with type II diabetes

Name	*n*	Median FU, years	CKD, %	HF, %	Primary composite outcome, HR (95% CI)	Notable secondary outcomes, RR reduction or HR (95% CI)	Adverse effects vs. placebo	Key findings
**EMPA-REG OUTCOME^158^**	7.020	3.1	25.9	10.1	CV death, non-fatal MI, non-fatal stroke: 0.86 (0.74–0.99)	All-cause mortality: −26%, *P*<0.01 HHF: −35%, *P*=0.002; Incident or worsening nephropathy^a^: HR 0.61 (0.53–0.70) Progression to macroalbuminuria: −34%, *P*<0.001	Male urinary tract infection, *P*<0.05 Genital infection, *P*<0.001 Acute renal failure, *P*<0.01	Empagliflozin reduced CV events in T2DM+ASCVD; signal in HF and CKD
**CANVAS Program^159^**	10.142	2.4	21.9	14.4	CV death, non-fatal MI, or stroke: 0.86 (0.75–0.97)	Progression of albuminuria: −31%, *P*<0.05	Amputation, *P*<0.001 Fracture, *P*<0.05 Male urinary tract infection, *P*<0.001 Female mycotic genital infection, *P*<0.001 Volume depletion, *P*<0.01	Canagliflozin reduced CV events in T2DM+ASCVD or at risk of ASCVD; signal in HF and CKD
**DECLARE-TIMI 58^157^**	17.160	4.2	7.4	10.0	CV death or HHF: HR 0.83 (0.73–0.95) MACE: 0.93 (0.84–1.03)	All-cause mortality: −7%, *P*>0.05 Renal composite^b^ or CV death: −24%, *P*<0.05	Major hypoglycaemia, *P*<0.05 Diabetic ketoacidosis, *P*<0.05 Acute kidney injury, *P*<0.01 Genital infection, *P*<0.001 Bladder cancer, *P*<0.05	Dapagliflozin reduced CV death or HHF, but had no effect on MACE, in T2DM+ASCVD or at risk of ASCVD
**VERTIS CV^160^**	8246	3.5	21.8	23.4	CV death, non-fatal MI, or stroke: 0.97 (0.85–1.11), *P*<0.001 for non-inferiority	HHF: −31%, *P*=0.006 Renal composite^c^: −18%, *P*=0.08	Urinary tract infection, *P*<0.05 Genital mycotic infection, *P*<0.001	Ertugliflozin was non-inferior but not superior to placebo in lowering CV events in T2D+ASCVD; signal in HF and CKD
**SCORED^175^**	10584	2	100	31	Total no. of CV deaths, HHF, and urgent visits for HF: 0.74 (0.63–0.88)	First occurrence of MACE: 0.84 (0.72–0.99) Total no. of HHF and urgent visits for HF: 0.67 (0.55–0.82) CV death −9%, *P*=0.35	Diarrhoea, *P*<0.001 Volume depletion, *P*<0.01 Genital mycotic infection, *P*<0.001 Diabetic ketoacidosis, *P*<0.05	Despite early stop of the trial, sotaglifllozin reduced HF events; signal in CV outcomes

ASCVD, ascertained atherosclerotic cardiovascular disease; CKD, chronic kidney disease; CV, cardiovascular; ESRD, end-stage renal disease; FU, follow-up; HF, heart failure; HHF, hospitalization for heart failure; T2D, Type 2 diabetes mellitus. ^a^eGFR<60 mL/min/1.73 m². ^b^>40% decrease in eGFR, ESRD, or death due to renal causes. ^c^Renal death, dialysis/transplant, and doubling of serum creatinine.

Based on encouraging data, the large DAPA-HF and EMPA-reduced trials were initiated in patients with or without T2D.^154^ With 4700 patients, DAPA-HF was the third largest study performed in the setting of HFrEF after PARADIGM-HF^166^ and SHIFT.^167^ Most recruited patients were in NYHA class II at the time of recruitment, with severe left-ventricular ejection fraction (LVEF) reduction (31% ± 6.7) and presence of ischaemic heart disease (57% of the total). Nearly half of patients had T2D at least one recovery for HF in their medical history. At baseline, all patients received guideline-directed medical therapy (GDMT). Dapagliflozin conferred an RR reduction of 26% as regards the primary outcome (the composite of CV death, recovery for HF, and urgent visit for worsening of HF) irrespective of the presence or absence of T2D, with a number needed to treat of 21. Shortly thereafter, with a similar design EMPEROR-Reduced presented the efficacy of empagliflozin in more advanced HFrEF patients with a mean age was 67 years, 3/4 in NYHA class II with a mean LVEF of 27%.^152,168^ Indeed, patients recruited had to be hospitalized for HF within 12 months, with higher levels of natriuretic peptides (doubled if concomitant atrial fibrillation). The primary outcome, CV death or HF hospitalization was reduced by 25% in the empagliflozin-treated arm. Yet, in contrast to dapagliflozin, empagliflozin failed to reduce CV death in HFrEF. Finally, the above-noted effects were independent of LVEF and T2D. Based on the results obtained in these landmark RCTs (*Table 2*) SGLT2i received an IA recommendation for the management of HFrEF patients.^9^

**Table 2.** Key clinical trials (in terms of findings and number of patients enrolled) of SGLT2i in patients with heart failure with (top) or without (bottom) reduced ejection fraction

Name	*n*	Median FU, years	T2D, %	CKD, %	Median EF, %	Median NT-proBNP, pg/mL	Primary composite outcome, HR (95% CI)	Notable secondary outcomes RR reduction or HR (95% CI)	Adverse effects vs. placebo	Key findings
***HFrEF***										
**DAPA-HF^162^**	4744	1.5	42	40.6	31.2±6.7	1428	CV death, HHF, or urgent HF visit: 0.74 (0.65–0.85)	Worsening renal function: −25%, *P*=0.17	None	Dapagliflozin reduced CV death and worsening HF events but not improved renal outcomes
**EMPEROR Reduced^165^**	3730	1.3	50	48	27.7±6.0	1887	CV death or HHF: 0.75 (0.65–0.86)	CV death: 0.92 (0.75–1.12) Renal composite^a^: 0.50 (0.32–0.77) All-cause mortality: −6%, *P*>0.05 Intensification of diuretics: −29%, *P*<0.001	Uncomplicated genital tract infections, *P*=N/A	Empagliflozin reduced HHF events, signal in renal outcomes and need for diuretic therapy intensification Higher prevalence of advanced HF compared with DAPA-HF
***HFpEF-HFmrEF***										
**Emperor-Preserved^169^**	5988	2.2	49	50	54.3±8.8 (34% with HFmrEF)	994	CV death or HHF: 0.79 (0.69–0.90)	CV death: 0.91 (0.76–1.09) Renal composite^b^: −3%, *P*>0.05	Uncomplicated genital tract infections, *P*=N/A Uncomplicated urinary tract infections, *P*=N/A Hypotension, *P*=N/A	Empagliflozin reduced HHF in patients hospitalized within 12 months
**DELIVER^170^**	6263	1.7	44.8	49.9	54.2±8.8 (34% HFmrEF)	1399	CV death or HHF or urgent visit HF: 0.82 (0.73–0.92)	Worsening HF: 0.79 (0.69–0.91) CV death: 0.88 (0.74–1.05)	None	Dapagliflozin reduced the combined risk with worsening HHF events leading the results
Mixed										
**SOLOIST-WHF^174^**	1222	0.75	100	70	35 (28–47) 29% HFpEF	1817	CV death, HHF, or urgent visit for HF: 0.67 (0.52–0.85)	CV death: −15%, *P*=0.36 Subgroup analysis: EF <40%: −21%, *P*=0.02 EF 40 to <50%: −37%, *P*=0.02 EF ≥50%: −37%, *P*=0.009	Severe hypoglycaemia, *P*=N/A	Despite early stop of the trial, sotagliflozin reduced HF events in patients with T2D who were recently hospitalized for worsening HF

CV, cardiovascular; ESRD, end-stage renal disease; EF, ejection fraction; FO, first occurrence; FU, follow-up; HF, heart failure; HFmrEF, heart failure with midrange ejection fraction; HFrEF, heart failure with reduced ejection fraction; HFpEF, heart failure with preserved ejection fraction; HHF, hospitalization for heart failure; T2D, Type 2 diabetes mellitus. ^a^Chronic haemodialysis, renal transplantation, and profound sustained reduction in eGFR. ^b^Time to first occurrence of chronic dialysis or renal transplant sustained reduction of eGFR.

The impressive results in the setting of HFrEF led to the design of innovative RCTs to test their efficacy in patients with HFpEF, a syndrome that was still lacking an effective medical therapy.^170,171^ Encouraging results were provided by EMPEROR-Preserved,^153^ in which older patients with LVEF >40% on baseline GDMT and a history of HF hospitalization within 12 months and NT-proBNP ≥300 pg/mL (or >900 pg/mL in presence of AF) were enrolled. The great majority was in NYHA class II with a mean LVEF of 54%. Nearly half had a diagnosis of T2D, AF, or CKD. In patients randomized to SGLT2i, a reduction of the primary outcome (composite of CV death or HF hospitalization) of 21% was observed, mainly driven by a 29% reduction of HF hospitalizations but with no significant benefit on CV death. Interestingly, the most prominent effect was observed in patients with LVEF ranging between 40 and 50%, a condition defined as HF with mid-range ejection fraction (HFmrEF).^9,11^ In those with an LVEF of ≥50 to <60% a borderline effect within a confidence interval of 0.64–0.99 was observed, while no effect was noted in those with a LVEF exceeding 60%. More recently, the DELIVER trial tested dapagliflozin specifically in patients with HFmrEF and HFpEF (LVEF ≥40%) over 2.3 years. Notably, this trial was the first to enrol also patients with HF with improved LVEF (HFimpEF), defined as HF with previously reduced LVEF of ≤40%. Overall, patients were mainly in NYHA class II with a median LVEF of 54% and hypertension and T2D as main comorbidities (89 and 44%, respectively).^155^ Dapagliflozin reduced the primary outcome (composite of worsening HF or CV death) by 18% (HR 0.82; 95%CI, 0.73–0.92). Yet, such reduction was mainly driven by the prevention of HF worsening (unplanned hospitalization for HF or an urgent visit for HF), while CV death, although lower in dapagliflozin arm (7.4 vs. 8.3%), did not reach statistical significance. Of interest, such results held true independent of LVEF and diabetes.^155^ Importantly, in patients with peripheral artery disease (PAD), a patient-level meta-analysis of DAPA-HF and DELIVER showed dapagliflozin to be highly effective in preventing worsening HF and CV death without increasing the risk of amputation as previously reported in the CANVAS trial.^172^ In both trials, the clinical benefits of dapagliflozin and empagliflozin in patients with HFmrEF and HFpEF occurred independent of the type and dosage of diuretics with a good safety profile allowing for a reduction of loop diuretic use over time.^173,174^ Based on these findings, EMPEROR-Preserved and DELIVER opened a new era for SGLT2i in HFmrEF and HFpEF as the first effective drug class for the treatment of these conditions.

Two more recent RCTs tested the combined SGLT1 and SGLT2 inhibitor sotagliflozin in individuals with T2D, i.e. SOLOIST-WHF and SCORED. In SOLOIST-WHF,^169^ sotagliflozin improved clinical outcomes in patients with T2D after an episode of decompensated HF independently of the presence or absence of HFrEF or HFpEF. Yet, this trial was not specifically designed to test HF population and recruited a moderate number of patients with this condition. In SCORED,^165^ sotagliflozin reduced the risk of CV death, HF hospitalization, or urgent visit for HF by 26%. Unfortunately, both trials were terminated prematurely due to loss of funding at the onset of the COVID-19 pandemic, with the number of HF patients constraining a subgroup analysis. However, a pooled analysis of both trials showed that sotagliflozin administration resulted in a significant reduction of CV death, hospitalization, or an urgent visit for HF, irrespective of LVEF over 9 to 16 months.^175^ In a subgroup analysis, patients with an LVEF of ≤40% experienced a 22% risk reduction, while patients with LVEF between 40 and 50% and patients with HFpEF showed a risk reduction of 43 and 33%, respectively.^176^ Based on the above, sotagliflozin recently received FDA approval for treatment of patients with HF independently of their LVEF.

One more smaller trial, i.e. EMPULSE^177^ recruited 530 patients with acute decompensated HF across the spectrum of LVEF that were randomized to empagliflozin or placebo, respectively. The primary analysis was based on the stratified win-ratio, focussing on the composite of death, number of events, time to first HF event, and change in Kansas City Cardiomyopathy Questionnaire-Total Symptom Score (KCCQ-TSS) from baseline to 90 days. Of note, clinical benefit was 14% higher in the empagliflozin group compared to the placebo arm. Notably, empagliflozin was also associated with fewer deaths and improvements in the quality of life.

Although not among the primary endpoints of EMPEROR-Preserved, DELIVER, and SOLOIST-WHF, consistent, time-dependent improvements in symptoms of HF as measured by KCCQ were provided by SGLTi. The primary endpoint of PRESERVED-HF,^178^ CHIEF-HF,^179^ and EMPERIAL-preserved trials,^179,180^ focused on improvement of HF symptoms, physical limitations, and exercise function in patients with HFpEF, both with or without T2D. In PRESERVED-HF the study population was older (mean age 69 years), the majority was in NYHA II, and around 50% had AF and/or T2D. The mean LVEF was 60% while mean NT-proBNP was 430 pg/mL (830 pg/mL in the presence of AF). In CHIEF-HF the mean age was 64 years, 1/3 were T2D with 40% having HFrEF. The primary outcome of both RCTs was the change in the KCCQ-TSS, although the study duration differed, with 3 and 12 months for CHIEF-HF and PRESERVED-HF, respectively. SGLT2i significantly improved quality of life at the end of the study period. In CHIEF-HF a reduction of 4.3 points vs. placebo irrespective of the type of HF or the presence of T2D was observed. Similarly, in PRESERVED-HF, KCCQ was reduced by six points. EMPERIAL-Preserved enrolled 315 patients with or without T2D and randomized them to empagliflozin or placebo for 12 weeks; however, the primary endpoint (6-min walk test distance change to week 12) did not differ between groups. Similarly, also in EMPERIAL-Reduced, empagliflozin did not provide substantial changes in 6-min walk test distance in patients with HFrEF.

In aggregate, most recent meta-analyses of the RCTs discussed above provide strong evidence of the efficacy of SGLT2i in preventing CV disease-related endpoints in patients with T2D and ability to reduce overall CV mortality by 15%.^156^ This effect is particularly evident in patients with ASCVD where CV death is reduced by 17%, while prevention of HF hospitalization (RR—30%) is consistently observed irrespective of ASCVD. A recent metanalysis in patients with HFrEF^158^ enrolled in DAPA-HF and EMPEROR-Reduced reported a reduction in CV death of 14% and of a first hospitalization for HF by 31%. Importantly, the composite outcome of CV death and HF hospitalization was reduced by 25% irrespective of the presence or absence of T2D. These beneficial effects remained robust despite different baseline characteristics such as age (absence of benefit only in those <55 years), sex, race, eGFR, NYHA class (major effects if NYHA II), history of HF hospitalization, or use of angiotensin receptor/neprilysin inhibitors (ARNI). As for patients with HFmrEF and HFpEF, a recent meta-analysis including data from DELIVER and EMPEROR-preserved showed a robust reduction of composite CV death or first hospitalization for HF [HR 0.80 (95% CI 0.73–0.87)].^8^ The same meta-analysis then included also data from DAPA-HF, EMPEROR-reduced, and SOLOIST-WHF to provide appropriate statistical power. When analysed together, the five trials further proved the efficacy of SGLT2i in preventing the risk of hospitalization for HF, extending survival, and improving overall health status. Such clinical benefits were consistent among 14 subgroups including age, sex, region, LVEF, T2D, kidney function, and body mass index.^8^ In contrast to other HF drugs, SGLT2i show an unusually rapid onset of action on clinical endpoints. Thus, together with the result of the STRONG-HF trial showing the safety and efficacy of a rapid up-titration of HF guideline-recommended therapies (yet without gliflozins),^181^ SGLT2i should be part of HFrEF therapy as soon as the diagnosis has been established.

### 4.2 SGLT2i in chronic kidney disease

When SGLT2i were first approved, the product label included warnings or even contraindications for their use in patients with reduced eGFR. Yet, consistent renoprotective effects were since observed in more recent trials with high-level evidence supporting their use in CKD. Surprisingly, their beneficial effects seem to be consistent in HF patients with CKD independently of the degree of renal impairment.^182^ T2D-related structural and functional alterations of the vascular endothelium, eventually leading to micro-albuminuria, are important contributors to adverse cardiorenal outcomes.^183,184^ Notably, SGLT2i consistently reduce albuminuria, attenuate the transition from micro-albuminuria to macro-albuminuria, and halt worsening renal function and thus the transition to ESRD,^185–187^ effects that act largely independent of changes in blood pressure, body weight, or HbA1c.^188,189^ In a prespecified analysis^190^ of the DAPA-CKD trial^191^ dapagliflozin increased the likelihood of regression to normo- or micro-albuminuria (with a 30% reduction of albuminuria after 2 weeks) and reduced the likelihood of progression to a high-degree albuminuria. As expected, patients with T2D showed greater benefit as compared to normoglycemic ones. Yet, the effects of SGLT2i were consistent across the spectrum of baseline eGFR and urinary albumin to creatinine ratio (UACR). Indeed, RCT-derived data suggest that SGLT2i blunt the development of ESRD, slow the decline in eGFR and reduce the risk of renal death while leading to an increase in erythropoietin production.^113^

Only few RCTs with SGLT2i explored renal outcomes as the primary endpoint (*Table 3*). In a meta-analysis of the EMPA-REG OUTCOME, CANVAS Program and DECLARE-TIMI 58 trials including a relatively heterogeneous study population in terms of eGFR (eGFR < 60 mL/min: 25.9% in EMPA-REG OUTCOME, 20.1% in CANVAS, and 7.4% in DECLARE-TIMI 58), SGLT2i consistently conferred renoprotective effects. The overall reduction of the composite endpoint of worsening renal function, ESRD, or renal death was 45% [HR 0.55 (95% CI 0.48–0.64)].^158^ This effect was similar both in patients with and without ASCVD (44 vs. 46%), while depending on baseline eGFR with greater benefit in patients with preserved compared to those with reduced renal function.

**Table 3.** Key clinical trials (in terms of findings and number of patients enrolled) of SGLT2i in patients with chronic kidney disease

Name	*n*	T2D, %	Median FU, years	Primary composite outcome, HR (95% CI)	Notable secondary outcomes	Adverse effects vs. placebo	Key findings
**CREDENCE^161^**	4401	100	2.6	ESRD, doubling of serum creatinine, renal or CV death: 0.70 (0.59–0.82)	All-cause mortality: −18%, *P*>0.05 CV death, MI, stroke, HHF/unstable angina: −34%, *P*<0.001	None	Canagliflozin improved renal outcomes in patients with T2D, signal in CV outcomes
**DAPA-CKD^192^**	4304	67.7	2.4	ESRD, eGFR reduction ≥50%, death from renal causes, CV death: 0.61 (0.51–0.72)	CV death/HHF: −29%, *P*<0.001 All-cause mortality: −31%, *P*=0.003	Major hypoglycaemia, *P*<0.05 Volume depletion, *P*=0.01	Dapagliflozin improved renal outcomes independently from T2D, signal in CV outcomes
**EMPA-KIDNEY^194^**	6609	46	3.1	ESRD, eGFR <10 mL/min/1.73 m^2^, eGFR reduction ≥40%, death from renal or cardiovascular causes: 0.72 (0.64–0.82)	Hospitalization for HF or cardiovascular death: 0.84 (0.67–1.07) Hospitalization for any cause: 0.86 (0.78–0.95)	None	Empagliflozin reduced kidney disease progression or death from cardiovascular causes

CV, cardiovascular; ESRD, end-stage renal disease; FU, follow-up; HF, heart failure; HHF, hospitalization for heart failure; T2D, Type 2 diabetes mellitus.

In a meta-analysis of DAPA-HF and EMPEROR-Reduced, the risk of the composite renal outcome (i.e. chronic dialysis, renal transplantation, or a ≥ 50% sustained reduction of eGFR) was significantly lower with SGLT2i, but to a lesser degree in the presence of CKD (23 vs. 27%, respectively). Of interest, changes in eGFR over time were similar in both trials.^7^ However, it must be noted that compared with those enrolled in DAPA-HF, patients included in EMPEROR-Reduced had consistently lower LVEF (27 vs. 31%, respectively), higher levels of natriuretic peptides, lower eGFR, and were more likely to have been treated with ARNI at baseline (20 vs. 11%, respectively). Very recently, another meta-analysis investigated the potential renoprotective effects of SGLT2i in 90 409 patients of 13 trials,^159^ further supporting the protective effect of SGLT2i with a 37% risk reduction of CKD progression, irrespective of the presence of T2D.

Potential renoprotective effects of SGLT2i among patients with HFpEF were hitherto inconsistent; indeed, no significant differences were observed in EMPEROR-Preserved with respect to renal outcomes. Similarly, a prespecified analysis of the DELIVER trial, while confirming the beneficial effect of dapagliflozin independently of baseline kidney function, did not show any effect of the drug on the renal composite outcome (an overall low event rate should be considered).^193^ However, dapagliflozin reduced the rate of decline in eGFR as compared with placebo.^193^

The CREDENCE trial recruited patients with T2D-related CKD with an estimated eGFR of 30 to <90 mL/min, a ratio of albumin to creatinine (UACR) exceeding 300 mg/g.^164^ The primary outcome was a composite of ESRD (dialysis, transplantation, or a sustained estimated GFR of <15 mL/min), a doubling of the serum creatinine level, or death from renal or CV causes. The trial was stopped early after a median of 2.6 years due to overwhelming benefit (−30% RR reduction of the primary outcome in the canagliflozin arm), with beneficial effects being noted irrespective of baseline HbA1c.

Although there seems to be a clear benefit of SGLT2i in improving cardiorenal outcomes among individuals with T2D, data from euglycemic population were scarce. DAPA-CKD aimed to fill this gap by enrolling patients with an eGFR 25–75 mL/min. and UACR of 200–5000 mg/g.^191^ Similar to CREDENCE, the trial was stopped early after 2.4 years when an interim analysis showed a clear benefit of dapagliflozin with an RR reduction of 39% of the primary outcome (i.e. composite of a sustained decline in the eGFR of at least 50%, ESRD, or death from renal or CV causes). The beneficial effects of dapagliflozin were again independent of the presence or absence of T2D, with a reduction in the mortality risk of 31% in the dapagliflozin group. Also, a recent post-hoc analysis of DAPA-CKD showed that treatment with dapagliflozin reduces anaemia and anaemia risk in patients with CKD independently of T2D. Finally, the largest phase III trial evaluating the effects of empagliflozin in adults with CKD independently from T2D (EMPA-KIDNEY) was stopped early due to an overwhelming renoprotective effect compared to placebo.^192^ In this trial, recruited patients had to be on GDMT with RAAS inhibitors and a eGFR between 20 and 45 mL/min or between 45 and 90 mL/min with UACR ≥200 mg/g (or protein/creatinine ratio ≥300 mg/g). After 2 years of follow-up, the primary endpoint (i.e. a composite progression of kidney disease or death from CV causes) was met by 13.1% of patients in the empagliflozin arm and by 16.9% of those allocated to placebo (HR 0.72; 95%CI 0.64 to 0.82). Results were independent of T2D and consistent among subgroups defined according to eGFR ranges.^192^

### 4.3 Gliflozins in myocardial infarction, atrial fibrillation, and cerebrovascular diseases

Hitherto, limited evidence exists on the effects of SGLT2i on myocardial infarction (MI)-related outcomes. The EMMY trial was among the first to assess whether empagliflozin in addition to guideline-recommended post-MI therapy leads to a larger decline in *N*-terminal pro-hormone of brain natriuretic peptide (NT-proBNP) and improvement in LVEF. Interestingly, among those receiving the active drug, a greater NT-proBNP reduction over 26 weeks together with improvements in cardiac structure and function were observed.^194^ These results are in line with observations in patients with ST-segment elevation MI (STEMI) showing a reduced cumulative incidence of MACE, HF, non-fatal MI, and unplanned repeat revascularization when patients were treated with dapagliflozin for other reasons.^195^ In November 2023 results of DAPA-MI, the first trial exploring the effect of dapagliflozin in addition to standard of care in patients with acute MI (and no T2DM), were made public.^196^ In this landmark trial, patients were randomly assigned to receiving placebo or dapagliflozin within 10 days from MI and impaired left ventricular (LV) systolic function or presence of Q-waves. The primary outcome (hierarchical composite of death, HF hospitalization, non-fatal MI, atrial fibrillation/flutter event, T2DM, New York Heart Association class IV?, weight decrease of ≥5%) was reached with a win-ratio of 1.34 (95%CI 1.20–1.50, *P* < 0.001). Interestingly, dapagliflozin-mediated clinical benefits were mainly driven by metabolic (T2DM and weight loss) rather than direct CV effects. Further results are expected soon from EMPACT-MI (NCT04509674), analysing the effect of empagliflozin in patients with acute MI and enrolling a slightly different patient population (including patients with T2DM and extending the enrolling period up to 14 days after MI). Furthermore, the primary endpoint of the ongoing EMPACT-MI trial differs markedly from that of DAPA-MI (i.e. composite one of the times to first HF hospitalization or all-cause mortality).

Patients with CV disease or T2D are prone to develop AF. Early preclinical evidence suggests a potential role for SGLT2i in preventing supraventricular arrhythmias with the underlying mechanisms still to be defined. Two recent meta-analyses comprising >30 studies support a reduction in AF risk of 19% and a 25% lower risk of serious AF-related events with SGLT2i.^197,198^ Such an effect appears to be particularly pronounced for dapagliflozin. In line, a recent post-hoc analysis of DECLARE-TIMI-58 showed beneficial effects of dapagliflozin on the incidence of AF with an RR reduction of 29%.^199^ Yet, a recent meta-analysis of EMPA-REG OUTCOME, DECLARE-TIMI 58, CANVAS Program, and CREDENCE found no clear effect of SGLT2i on cerebrovascular events; however, there was some benefits as it relates to haemorrhagic stroke and AF, as well as total stroke for those reduced eGFR.^200^ When interpreting such results one should bear in mind that none of the RCTs on SGLT2i available to date was designed to investigate AF-related outcomes. Furthermore, AF occurrence is not routinely reported in the primary analysis of most SGLT2i RCTs, often lacking follow-up data on electrocardiograms. Indeed, most trials only report AF events meeting the criteria for serious adverse events; as a result, available RCTs showed a lower than that expected AF event rate in such a high-risk population compared to real-world data.^201^ A specific trial on dapagliflozin (DAPA-AF; NCT04792190) is designed to investigate the effect of the drug on AF burden are expected to provide new evidence.

Patients with established CV diseases or a diagnosis of T2D are at high risk of ischaemic stroke.^202^ However, with stroke being a relatively rare event coupled with the relatively short observation periods, most RCTs were inconclusive when it comes to the effects of SGLT2i on stroke-related outcomes. Nonetheless, previous meta-analyses suggest that SGLT2i may have a neutral effect on the risk of stroke in patients with T2D.^160–162^ A more recent meta-analysis discerning the effects on the different stroke subtypes reported a possible beneficial effect on haemorrhagic stroke over placebo, which might be related to blood pressure reduction provided by SGLT2i. Yet, due to the low number of events such findings warrant confirmation in well-designed RCTs.^203^

## 5. Future perspectives and conclusions

Gliflozins had a remarkable impact on the management of both T2D and its clinical sequalae. Indeed, SGLT2i represent the first antidiabetic drug class shown to effectively reduce HF hospitalizations, CV death, and worsening kidney function in specific patient populations. Their CV benefits as well as the underpinning mechanisms are multifaceted, with their cardioprotective effects acting independently of their glucose-lowering properties thereby providing beneficial effects beyond glycaemic control. While their CV benefits involve cellular and molecular mechanisms at systemic, vascular, cardiac, and renal levels, their cardiorenal effects appear to contribute to their clinical benefits most prominently. As data on their protective effects irrespective of LVEF are accumulating, international societies have recently expanded their recommended use to the entire spectrum of HF. With roughly 50 phase III RCTs presently ongoing (clinicaltrials.gov; accessed on 19 December 2023; *Table 4*), SGLT2i—also referred to as ‘statins of the 21st century’^204^—are likely to assume additional applications in different fields and medical conditions in the near future. Potential beneficial effects of gliflozins in patients with acute HF, atrial fibrillation, valvular heart disease, cardiogenic shock, and hypertrophic cardiomyopathy, among others, are currently being investigated. Beyond cardiology, other disciplines including nephrology (eg, CKD) and hepatology (eg, non-alcoholic fatty disease) may benefit from the rigorous study of this class of drugs at both experimental and clinical levels.

**Table 4.** Ongoing phase III randomized clinical trials on SGLT2i exploring cardiovascular, cerebrovascular, or renal outcomes in different conditions

Trial number	Treatment	Disease/condition	Primary outcome
NCT06142474	Dapagliflozin or empagliflozin on top of standard care	Acute decompensated HF during ventilator weaning	Composite: weaning failure, recurrent pulmonary oedema, and CV/non-CV mortality
NCT05345327	Dapagliflozin vs. metformin	Renal function decline in type II diabetes	Rate of decline in eGFR
NCT04965935	Dapagliflozin on top of standard care	Renal transplantation	Systolic blood pressure
NCT05468203	Dapagliflozin vs. placebo	Acute kidney injury in intensive care	Composite: doubling of serum creatinine from baseline, initiation of renal replacement therapy or death
NCT05241431	Dapagliflozin vs. placebo	Aortic valve stenosis intervention (TAVR)	Composite: changes in LV mass, systolic function, eGFR, and serum NT-proBNP
NCT05776043	Empagliflozin or dapagliflozin vs. placebo	Acute decompensated HF	Composite: CV death, hospitalization for HF in patients with HFrEF
NCT04298229	Dapagliflozin on top of standard care	Acute decompensated HF	Cumulative change in weight
NCT05373004	Empagliflozin vs. enalapril maleate	Renal function decline in type II diabetes	Rate of decline in eGFR
NCT05310916	Dapagliflozin on top of standard care	Diabetic retinopathy	Severity of retinopathy
NCT05373680	Empagliflozin vs. metformin	Chronic kidney disease	Rate of decline in eGFR
NCT06024746	Finerenone+empagliflozin vs. usual care	Hospitalized patients with HF	Hierarchical composite of the following assessed by the win-ratio: -Time to death from any cause -Number of HF events -Time to first HF event -Difference of KCCQ-TSS
NCT03723252	Dapagliflozin vs. placebo	Non-alcoholic steatohepatitis	Liver histological improvement
NCT05139472	Empagliflozin	HFpEF	Peak oxygen uptake
NCT04049110	Dapagliflozin vs. placebo	Type I diabetes	Mean amplitude of glucose excursions after physical exercise
NCT03215069	Empagliflozin vs. placebo	Gestational diabetes	Baseline-adjusted ISSI-2
NCT03717194	Ertugliflozin vs. placebo	Type II diabetes and HF	Change of global longitudinal strain
NCT06124937	Empagliflozin vs. placebo	Post-operative atrial fibrillation	Incidence of post-operative atrial fibrillation
NCT05321706	Dapagliflozin vs. placebo	Renal function decline in heart transplant	Rate of decline in eGFR
NCT04231331	Ertuglifluzin vs. placebo	Functional mitral regurgitation	Change of effective regurgitant orifice area
NCT05879276	Empagliflozin on top of standard management	Cardiogenic shock	Hierarchical composite of the following assessed by the win-ratio -Time to all-cause death or cardiac transplantation or mechanical ventricular assist -Time to rehospitalization for HF -Left ventricular ejection fraction assessed during a research cardiac ultrasound
NCT05182658	Empagliflozin vs. placebo	Hypertrophic cardiomyopathy	Change in peak VO2 measured in the cardiopulmonary exercise testing
NCT05764057	Dapagliflozin vs. placebo	Post-ischaemic cardiac remodelling	-Change in left ventricular ejection fraction -Change in atrial volume
NCT05580510	Empagliflozin, sacubitril/valsartan, or their combination on top of standard management	HFrEF in adult congenital heart disease	-Systemic ventricular end-diastolic volume index -Systemic ventricular end-systolic volume index
NCT06027307	Enavogliflozin vs. placebo	Functional tricuspid regurgitation in HFpEF	Composite of CV death, hospitalization for HF, or worsening of tricuspid regurgitation
NCT05998525	Dapagliflozin vs. placebo	Coronary artery disease after acute myocardial infarction	-Changes in the coronary calcium score -Changes in cardiac epicardial fat volume
NCT06103279	Empagliflozin vs. placebo	Doxorubicin-induced cardiomyopathy	-Cardiac troponin T -LVEF
NCT05556044	Empagliflozin on top of standard management	New-onset HF	-Cumulative of hospitalization for HFs, urgent HF visits, and unplanned outpatient visits -All-cause mortality
NCT05271162	Empagliflozin vs. placebo	Anthracycline-induced cardiomyopathy	LV systolic dysfunction
NCT04509674	Empagliflozin vs. placebo	Myocardial infarction	Composite of time to first HF hospitalization or all-cause mortality
NCT04393246	Dapagliflozin and ambrisentan on top of standard management	COVID-19	Composite endpoint of: death, mechanical ventilation, ECMO, CV organ support, or renal failure
NCT06174753	Dapagliflozin vs. placebo	STEMI	Infarct size
NCT06111443	Dapagliflozin on top of standard management	Catheter ablation of atrial fibrillation	Freedom from all atrial tachyarrhythmias
NCT06030843	Empagliflozin vs. placebo	Cardiorenal syndrome Type 1	Composite outcome of death, new dialysis, and sustained loss of kidney function
NCT06000462	Dapagliflozin vs. metformin vs. placebo	Obesity	Reduction in baseline weight
NCT05957887	Dapagliflozin on top of standard management	Anterior STEMI	Global longitudinal strain
NCT05944016	Dapagliflozin vs. placebo	Chronic kidney disease in adolescents and young adult	Change in urine albumin to creatinine ratio
NCT05905965	Empagliflozin 10 vs. 20 mg	Metabolic syndrome	-HbA1c -BMI
NCT05715814	Empagliflozin on top of standard management	Peritoneal dialysis	Change in measured GFR
NCT05618223	Dapagliflozin on top of standard management	Rheumatic mitral atenosis	-Biomolecular parameters (PICP, MMP-1, MMP-1/TIMP-1 ratio, TGF-β, NT-proBNP) -Clinical parameters (KCCQ score) -Echocardiography parameters (net atrioventricular compliance, mitral valve gradient, and mPAP)
NCT05558098	Dapagliflozin on top of standard management	Critically ill patients	Hierarchical composite of the following assessed by the win-ratio: -hospital mortality -use of kidney replacement therapy -intensive care unit length of stay
NCT05392764	Empagliflozin vs. placebo	Acute HF	Hierarchical composite of the following assessed by the win-ratio: -death within 90 days -HF rehospitalization within 90 days -worsening HF during hospitalization -urine output up to 48 h after treatment initiation
NCT05364190	Canagliflozin vs. empagliflozin	Acute HF	Cumulative mean of daily diuresis
NCT05196347	Dapagliflozin on top of standard management	Advanced chronic kidney disease (Stage 4 and 5)	eGFR decline
NCT04792190	Dapagliflozin vs. placebo	Atrial fibrillation	Change in burden of atrial fibrillation

CV, cardiovascular; ECMO, **e**xtracorporeal membrane oxygenation; eGFR, estimated glomerular filtration rate; HFrEF, heart failure with reduced ejection fraction; HFpEF, heart failure with preserved ejection fraction; ISSI-2, insulin secretion sensitivity index-2; KCCQ, Kansas City cardiomyopathy questionnaire; LV, left ventricule; MMP, matrix metalloproteinase; mPAP, mean pulmonary arterial pressure; NT-proBNP, N-terminal pro B-type natriuretic peptide; PICP, procollagen I C-terminal propeptide; STEMI, ST-elevation myocardial infarction; TAVR, transcatheter aortic valve replacement; TGF, transforming growth factor; TIMP, tissue inhibitor of MMP.
